# Inflammation in Retinal Disease 

**DOI:** 10.1155/2013/724648

**Published:** 2013-09-10

**Authors:** Scott M. Whitcup, Robert B. Nussenblatt, Susan L. Lightman, David A. Hollander

**Affiliations:** ^1^Allergan, Inc., Irvine, CA 92623-9534, USA; ^2^Laboratory of Immunology, National Eye Institute, National Institute of Health, Bethesda, MD 20892-2510, USA; ^3^UCL Institute of Ophthalmology, Moorfields Eye Hospital, London EC1V 2PD, UK

## 1. Introduction

Ocular inflammation and its related complications are important causes of vision loss. Inflammatory processes have long been implicated in the pathogenesis and sequelae of noninfectious uveitis and understood to underlie the macular edema which may arise following even uncomplicated intraocular surgeries [1]. More recently, evidence has also arisen supporting a prominent role for inflammation underlying the pathogenesis of a wide array of retinal diseases, including age-related macular degeneration (AMD) [2], diabetic retinopathy (DR) [3], retinal vein occlusion (RVO) [4], and retinitis pigmentosa (RP) [5], and has suggested a role for anti-inflammatory therapies to potentially alter the severity and course of these disorders. The goal of this special issue is to highlight the latest understanding of the role of inflammation in retinal diseases, to address current questions and controversies, and to facilitate future research.

Traditionally, the eye has been considered an immune privileged site. Contributing to this immune privilege is the blood-retinal barrier which consists of both an inner barrier formed by the tight junctional complexes between retinal vascular endothelial cells and an outer barrier formed by the tight junctions between the retinal pigment epithelium (RPE) cells. Research over the last 30 years has demonstrated that mechanisms beyond tissue barriers contribute to ocular immune privilege and an immunosuppressive intraocular environment. In fact, the pigment epithelial cells which line the iris, ciliary body, and retina serve an immunomodulatory role through both the secretion of soluble immunosuppressive factors as well as contact-dependent mechanisms [6]. 

Vision is dependent on the exquisite and precise structure of the retina, and any process which significantly disrupts retinal architecture can have a profound impact on vision. The immune response, when controlled, is an adaptive response to restore homeostasis. Alterations in retinal homeostasis secondary to aging, metabolic abnormalities, altered vascular perfusion, or degenerative genetic conditions may initiate various inflammatory cascades. In all of these settings, a prolonged, dysregulated immune response may itself be pathologic, contributing to both the pathogenesis of retinal diseases as well as vision threatening complications. 

## 2. Age-Related Macular Degeneration

Age-related macular degeneration (AMD) is a leading cause of irreversible vision loss in the western world. AMD can manifest as both a “dry” form (90% of cases) featuring geographic atrophy of the RPE which currently has no treatment as well as an exudative “wet” form (10% of cases) which is responsible for the majority of cases of vision loss due to choroidal neovascularization which may now respond to treatment with antivascular endothelial growth factor (VEGF) agents. While genetic, environmental, and metabolic factors may all be contributing factors, recent evidence supports a more central role for the immune system in the pathogenesis of AMD. 

Aging is associated with a decrease in the number of RPE cells as well as the number of photoreceptors [7]. With aging, oxidative stress secondary to the accumulation of oxidized lipoproteins and free radicals in retinal and choroidal tissues may trigger a tissue adaptive response, recently described as “para-inflammation,” in which cells of the innate immune system mount a low-grade inflammatory response in order to restore tissue homeostasis [8]. Sustained injury or chronic inflammation may lead to an imbalance in the local inflammatory response and contribute to AMD. 

Drusen, extracellular deposits located between the RPE and Bruch's membrane, are most commonly seen in individuals over 60 years of age and represent the clinical hallmark of AMD. Though once considered simply to be waste products consisting of lipid and carbohydrate, drusen are now understood to also consist of byproducts of local active inflammation and complement activation (C3, C5a, and C9) [9]. The etiology of drusen and the progression of AMD are likely multifactorial, though a primary mechanism may be related to RPE cell injury. Injured RPE cells release cytokines and chemokines that recruit and activate choroidal dendritic cells. Dendritic cells may amplify the inflammatory process via cell to cell contact, immune complex formation, and complement activation leading to additional RPE cell damage, potentially producing a state of chronic inflammation [9]. 

In addition to elements of the complement system being found in drusen, both genetic and animal studies have also strongly supported a pivotal role of the complement system in the pathogenesis of AMD. While complement is active at a low basal level in the normal retina as a protective mechanism, alterations in the regulation of the complement system can trigger significant pathology. Strong associations with AMD have been identified in association with particular mutations in the complement factor H (CFH) protein (Y402H), a key regulatory component of the alternative pathway in distinguishing self from nonself [10–13]. Thus, a growing body of histopathological, preclinical, and epigenetic data now supports a key role of inflammation in the pathogenesis of AMD, a disease which was not classically described as inflammatory in origin.

## 3. Diabetic Retinopathy

Over 285 million individuals worldwide are estimated to have diabetes mellitus [14]. Diabetic retinopathy (DR), a common complication of diabetes, increases in prevalence with duration of disease. DR has traditionally been considered a disease of the retinal microvasculature and has been categorized into an early nonproliferative stage and an advanced, proliferative stage based on the natural history. The most common causes of vision loss in diabetics are diabetic macular edema (DME), typically seen early in the course of DR, and proliferative retinopathy. 

The mechanisms by which high glucose levels directly lead to diabetic retinopathy have not been fully elucidated. Chronic hyperglycemia leads to a series of biochemical changes, including activation of protein kinase C, accumulation of polyols through the aldose reductase pathway, increased formation of advanced glycation end products (AGEs), and overproduction of free radicals. These metabolic changes increase proinflammatory cytokines, chemokines, and other inflammatory mediators that stimulate an influx of leukocytes and alter vascular permeability [15]. Elevated levels of interleukin 6 (IL-6), IL-8, tumor necrosis factor-*α* (TNF*α*), VEGF, interferon-induced protein-10 (IP-10), intercellular adhesion molecule 1 (ICAM-1), and monocyte chemoattractant protein-1 (MCP-1) have been demonstrated in eyes with DR [15]. 

Inflammatory processes may underlie many of the functional retinal vasculature alterations observed histologically in early diabetic retinopathy, such as pericyte loss, saccular microaneurysms, and occluded and degenerated capillaries. An increase in the attraction and adhesions of leukocytes has been observed in experimental models of diabetes within 1 week of disease onset [16]. This leukostasis is a direct result of the interactions between elevated expression of ICAM-1 on retinal vessels and the CD18 adhesion molecule on monocytes and neutrophils [16]. Increased leukocyte stiffness may also contribute to capillary nonperfusion [17]. Experimental models of diabetes in mice deficient in the genes encoding for ICAM-1 and CD18 have revealed fewer adherent leukocytes in the retinal vasculature, a reduced number of damaged endothelial cells, and less vascular leakage [3]. 

In addition to the leukocyte-mediated endothelial cell damage, increased vascular permeability leading to DME also arises due to conformational alterations in the tight junctional proteins. The tight junctions consist of over 40 different proteins and various inflammatory mediators, including VEGF, TNF*α*, protein-kinase-C, IL-1*β*, and IL-6, alter particular proteins via phosphorylation, redistribution, or alteration in content thereby reducing the endothelial barrier [18]. As inhibition of different inflammatory mediators has been shown to limit the degeneration of retinal capillaries characteristic of early stages of DR, continued investigations into the role of inflammation in the pathogenesis of DR are warranted. 

## 4. Retinal Vein Occlusion

Retinal vein occlusion is the second most common ocular vascular abnormality, following diabetic retinopathy, resulting in vision loss. The occlusion may occur at or proximal to the lamina cribrosa of the optic nerve involving the central retinal vein or occur more commonly at an arteriovenous intersection involving a branch retinal vein. The origin of the occlusion likely stems from compression and local retinal vascular damage, followed by stasis and thrombosis. In some patients, inflammatory conditions may play a role in contributing to the vascular injury and thrombus formation [4]. Increased hydrostatic pressure proximal to the occlusion commonly leads to vascular leakage and subsequent macular edema, the most frequent cause of vision loss in the setting of RVO. 

Vascular endothelial damage in the occluded vein may result in a low-grade, chronic inflammation and the production of inflammatory mediators that exacerbate and prolong the edema. A number of inflammatory cytokines and growth factors may be elevated in RVO patients, including IL-1*α*, IL-6, IL-8, MCP-1, platelet derived growth factor (PDGF-) AA, and VEGF relative to control eyes [19–21]. These factors contribute to the transition from an acute to chronic inflammation, the recruitment of monocytes to the site of injury, an increase in vascular permeability, and the development of ocular neovascularization. The severity of macular edema secondary to BRVO has been correlated with both elevated vitreous and aqueous levels of VEGF and IL-6 [22]. 

## 5. Retinitis Pigmentosa

Retinitis pigmentosa is a heterogeneous group of inherited retinal degenerative diseases which lead to photoreceptor cell death and severe vision loss. Clinically, RP is characterized by a pigmentary retinopathy, optic nerve pallor, progressive visual field loss, and nyctalopia. Additional clinical findings may include vitreous cells, posterior subcapsular cataract, and macular edema. Lymphocytes have been detected in the vitreous gel of RP patients, further characterizing the inflammatory nature of the vitreous cells [23]. While RP is now known to be primarily a hereditary disease caused by mutations in over 45 different genes, investigators have continued to examine the role of the immune system in the pathogenesis and progression of the disease. 

It has been suggested that the observed immune responses are likely secondary to the release of retinal proteins by the underlying degenerative disease [24]. First, major differences in immune responses have not been detected across different subtypes of RP [24]. Secondly, it has often been in those patients with severe vision loss that significant cellular immune responses have been shown [25]. In a recent clinical study of RP patients, greater inflammation in the anterior vitreous correlated with worse VA as well as lower mean deviation on visual field testing [5]. Elevated proinflammatory markers, most notably MCP-1, have been detected in both the aqueous and vitreous [5]. MCP-1 is known to activate microglia as well as recruit monocytes, memory T cells, and dendritic cells to sites of injury. While the chronic inflammation in RP patients may be secondary to a primary genetic mutation leading to photoreceptor loss, the immune response to the shed proteins may subsequently exacerbate the retinal destructive processes in RP and other retinal degenerative diseases [26]. 

## 6. Conclusion

We believe that the papers included in this issue will offer readers a greater appreciation for the role of inflammation in a variety of retinal diseases, many of which were not traditionally considered to be inflammatory in nature. 

Whitcup et al. summarize discussions from the 5th annual conference of the Arnold and Mabel Beckman Initiative for Macular Research by the Inflammation and Immune Response Task Force in which they review data supporting the dysregulation of immune response as a contributing factor to the pathogenesis of AMD and propose a series of experimental approaches to address unanswered questions. 

In a mouse model of AMD, Cruz-Guilloty et al. demonstrate a link of AMD-like histopathological changes with the presence of macrophages in the outer retina during early stages of disease. The authors suggest that immune modulation may play a role in the future in either the prevention or treatment of patients with early signs of AMD. 

Jain et al. address the evolving pharmacologic treatment options for DME, focusing on the multifactorial nature of the disease in their review of major studies of both corticosteroids and anti-VEGF therapies. 

Deobhakta and Chang summarize the laboratory and clinical studies supporting the role of inflammation in the pathogenesis and clinical consequences of RVO. The authors also review the latest clinical studies of anti-inflammatory treatments for patients with macular edema secondary to RVO. Using ultra wide field fluorescein angiography, Tsui et al. report late peripheral retinal leakage in the fellow eyes in patients with BRVO and suggest that these findings may represent underlying systemic inflammation, hypertension, or bilateral BRVO. 

Viringipurampeer et al. review the preclinical and clinical evidence linking inflammatory mediators to genetic retinal diseases, specifically RP and AMD, and summarize the latest anti-inflammatory interventional studies. The authors conclude that anti-inflammatory agents are likely to play significant roles in the future treatment algorithms of these diseases. 

Schoenberger and Kim review the role of nonsteroidal anti-inflammatory drugs (NSAIDs) as inhibitors of the cyclooxygenase (COX) enzymes that catalyze the synthesis of prostaglandins. The authors review the scientific rationale and provide an update on the interventional studies that have been conducted with NSAIDs in postoperative cystoid macular edema, AMD, DME, and DR. 

The ideas discussed in this issue should demonstrate that immune responses, while often beneficial in the acute setting, can have undesirable effects if they result in a state of chronic inflammation. Ultimately, as the roles of different inflammatory pathways in retinal diseases become more clearly elucidated, greater emphasis can be placed on new targets for future treatment options.

We would like to dedicate this special issue to Stephen J. Ryan, MD, who passed away on April 29, 2013. Dr. Ryan was an expert in retinal diseases and a leader in ophthalmology. He was the president of the Doheny Eye Institute from 1974 to 2012, the first full-time chairman of the University of Southern California (USC) Department of Ophthalmology, and the dean of USC's school of medicine from 1991 to 2004 which later became the Keck School of Medicine. Dr. Ryan also was a member of the Institute of Medicine and a member of the National Advisory Eye Council and founded the National Alliance for Eye and Vision Research (NAEVR). Dr. Ryan devoted his career to understanding the pathogenesis of diseases of the retina including age-related macular degeneration. In addition to his own pioneering research, Dr. Ryan trained and educated countless scientists and clinicians around the world. It is therefore befitting that we dedicate this collection of manuscripts discussing the role of inflammation on the pathogenesis of retinal diseases to Dr. Ryan.

*Scott M. Whitcup* *Scott M. Whitcup* *Robert B. Nussenblatt* *Robert B. Nussenblatt* *Susan L. Lightman* *Susan L. Lightman* *David A. Hollander* *David A. Hollander*
